# Three-dimensional growth: a developmental innovation that facilitated plant terrestrialization

**DOI:** 10.1007/s10265-020-01173-4

**Published:** 2020-02-24

**Authors:** Laura A. Moody

**Affiliations:** Department of Plant Sciences, University of Oxford, South Parks Road, Oxford, OX1 3RB UK

**Keywords:** Apical growth, Brown algae, Charophyte, Development, Evolution, Land plant, Three-dimensional

## Abstract

One of the most transformative events in the history of life on earth was the transition of plants from water to land approximately 470 million years ago. Within the Charophyte green algae, the closest living relatives of land plants, body plans have evolved from those that comprise simple unicells to those that are morphologically complex, large and multicellular. The Charophytes developed these broad ranging body plans by exploiting a range of one-dimensional and two-dimensional growth strategies to produce filaments, mats and branches. When plants were confronted with harsh conditions on land, they were required to make significant changes to the way they shaped their body plans. One of the fundamental developmental transitions that occurred was the evolution of three-dimensional growth and the acquisition of apical cells with three or more cutting faces. Plants subsequently developed a range of morphological adaptations (e.g. vasculature, roots, flowers, seeds) that enabled them to colonise progressively drier environments. 3D apical growth also evolved convergently in the brown algae, completely independently of the green lineage. This review summarises the evolving developmental complexities observed in the early divergent Charophytes all the way through to the earliest conquerors of land, and investigates 3D apical growth in the brown algae.

## Introduction

One of the most transformative events in the history of life on earth was the emergence of terrestrial plant life approximately 470 million years ago (Delwiche and Cooper 2015; Kenrick and Crane 1997). Before this time, photosynthesis only occurred in aquatic environments. Plants evolved a range of molecular, biochemical and morphological adaptations to enable them to survive and reproduce on land (Minami et al. 2003; Stevenson et al. 2016; Wolf et al. 2010). These included roots for anchorage and the acquisition of water and nutrients, leaves for capturing sunlight, vascular tissues for transport and mechanical support, and seeds and flowers for enhanced reproductive success. One of the most important innovations that enabled the assembly of these morphological traits, was the evolution of three-dimensional (3D) growth. 3D growth is an invariable and pivotal feature of all land plants, and the diverse sizes and morphologies exhibited across the terrestrial biosphere are all due to differential regulation of 3D growth processes during development. Interestingly, 3D growth is also exhibited within species of brown algae, and thus evolved completely independently of the green lineage in the water.

## The streptophytes

**Fig. 1:**
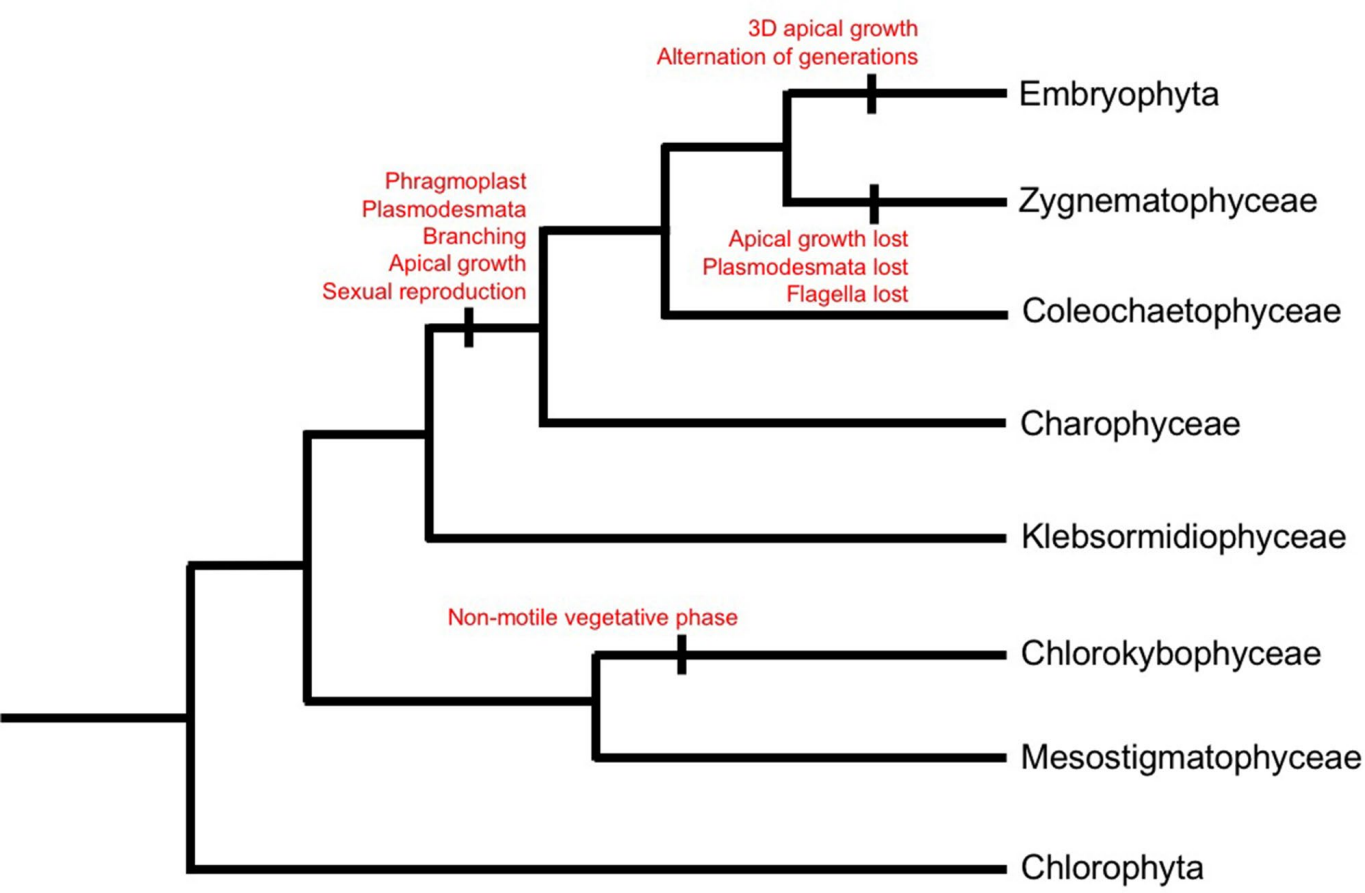
Evolution of the Streptophytes. Cladogram illustrating the evolution of the Charophytes and land plants (‘the Streptophytes’). The cladogram topology was selected based on recent phylogenetic studies that place the Zygnematophyceae as the closest living relative to land plants (Puttick et al. 2018; Timme et al. 2012; Wickett et al. 2014). The gains and losses of important morphological characteristics within the lineages have been indicated.

The Viridiplantae (‘green plants’) comprise the Chlorophyta (freshwater and marine algae) and the Streptophytes, which include the Charophyte group of green algae and the land plants (Lewis and McCourt 2004). The Charophytes, which reside in a range of freshwater and subaerial habitats, are the closest living relatives of land plants (Kenrick and Crane 1997; Leliaert et al. 2012). There are six distinct classes within the Charophytes; Mesostigmatophyceae, Chlorokybophyceae, Klebsormidiaceae, Coleochaetophyceae, Charophyceae and the Zygnematophyceae (McCourt et al. 2004). Some studies have positioned Charophyceae as the closest living relative of land plants, a reasonable assumption considering the morphological complexity within the group (Karol et al. 2001; Turmel et al. 2003). However, other studies have suggested that the Coleochaetophyceae or a combined Coleochaetophyceae/Zygnematophyceae clade represent the closest relatives of land plants (Finet et al. 2010; Wodniok et al. 2011). More recent and well supported phylogenetic studies place the Zygnematophyceae as the closest living relative to land plants (Fig. 1; Puttick et al. 2018; Timme et al. 2012; Wickett et al. 2014). As streptophyte lineages diverge, their morphological diversity becomes increasingly complex, from organisms that are unicellular to those that are multicellular non-branching to multicellular branching to those with complex multicellular organisation and subsequently those that exhibit 3D growth. This review summarizes the evolving morphological complexity within the Streptophytes, from early divergent Charophytes to the earliest divergent land plants.

### The early divergent charophytes

**Fig. 2:**
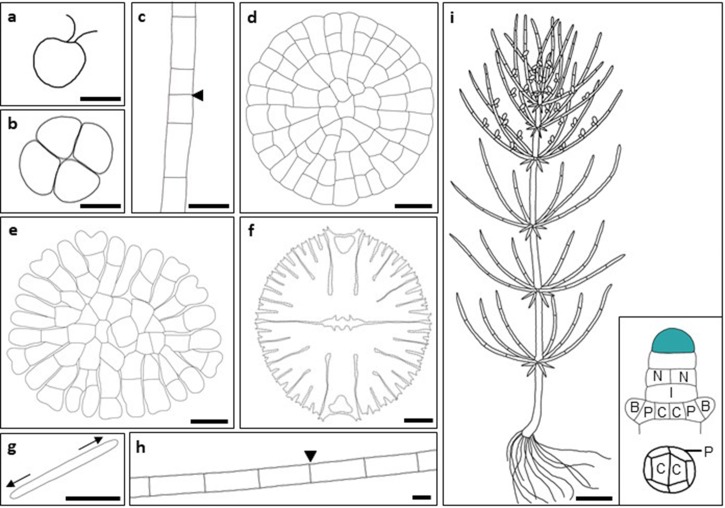
Representatives of the Charophytes. Schematic diagrams of cellular organisation in representatives from each of the Charophyte lineages. **a–c** The early divergent Charophytes are represented by the scaly unicellular flagellate *Mesostigma viride* (**a**), *Chlorokybus atmophyticus* (**b**) and multicellular, non-branching *Klebsormidium nitens* (**c**). **d**–**i** The late divergent Charophytes; *Coleochaete orbicularis* (**d**), *Coleochaete soluta* (**e**); *Micrasterias denticulata* (**f**); *Penium margaritaceum* (**g**); *Spirogyra pratensis* (**h**) and *Chara braunii* (**i**). The inset image in (**i**) shows the cellular organisation within the growing apex of *Chara braunii*. The top image shows that growth occurs through divisions of a terminal apical cell (green), which gives rise to alternating nodal (N) and multinucleate internodal (I) cells. Nodal cells divide several times vertically to produce two central (C) cells surrounded by a marginal row of peripheral (P) cells, which form the apical cells of radiating branches (B). The bottom image shows a transverse section through an established node. Arrowheads in (**c**) and (**h**) denote the most recent intercalary cell division. Arrows in (**g**) denote direction of growth. Images **d**–**f** and **i** were adapted from Graham et al. (2015) and Nishiyama et al. (2018) respectively. Scale bars 10 µm (**a**–**c**), 20 µm (**d**, **e**) 30 µm (**f**–**h**) and 1 cm (**i**).

The Mesostigmatophyceae and Chlorokybophyceae are widely accepted to represent the earliest diverging streptaphyte lineages, followed by the divergence of the Klebsormidiaceae (Lemieux et al. 2007; Rodriguez-Ezpeleta et al. 2006; Timme et al. 2012). The early divergent Charophytes divide by a distinct mechanism of cell division, which involves the formation of a cleave furrow (Leliaert et al. 2012). The Mesostigmatophyceae are considered the most basal group within the Charophytes. The representative species *Mesostigma viride* is defined as a scaly green unicellular flagellate, which has three layers of ornate scales in the absence of a cell wall (Fig. 2a). These unicells are typically asymmetrically shaped, bear two flagella and occupy freshwater habitats (Marin and Melkonian 1999; Rogers et al. 1981). The Chlorokybophyceae are represented by the sole species *Chlorokybus atmophyticus*, which forms packets of non-motile cells that reproduce asexually (Fig. 2b). *Chlorokybus atmophyticus* occupies subaerial habitats and has the capacity to survive desiccation, freezing and UV exposure (Lokhurst et al. 1988; Rogers et al. 1980). The Klebsormidiaceae are composed of multicellular and non-branching filaments with cell division taking place throughout the length of the filament, and not at apices (Fig. 2c; Pierangelini et al. 2017). Representatives of the Klebsormidiaceae can survive in water as well as subaerial conditions and are both desiccation and freezing tolerant (Elster et al. 2008; Morison and Sheath 1985; Nagao et al. 2008). The genome sequence of *Klebsormidium nitens* (formerly identified as *Klebsormidium flaccidum*) revealed that the Klebsormidiaceae produce phytohormones and may respond to radiation exposure using similar mechanisms to those found in land plants (Hori et al. 2014).

### The late divergent charophytes

The Coleochaetophyceae, Charophyceae and Zygnematophyceae share a number of key characteristics with land plants, such as the formation of complex cell walls, plasmodesmata for intercellular communication, and the ability to divide asymmetrically (Cook et al. 1997; Nishiyama et al. 2018; Sorensen et al. 2011; Umen 2014). The late divergent Charophytes and the land plants also established a new mechanism of cell division, which involved the formation of a phragmoplast (Cook et al. 1997). For this reason, the Coleochaetophyceae, Charophyceae, Zygnematophyceae and the land plants collectively form the ‘Phragmoplastophyta’ (Nishiyama et al. 2018).

The Coleochaetophyceae form branching filaments, in which apical cells with two cutting faces give rise to two-dimensional (2D) mats. *Coleochaete orbicularis* appears as unistratose discs, which grow by means of a marginal meristem. Cells within the thallus are generally constrained by neighbouring cells but peripheral cells are capable of polar expansion and grow away from the centre of the thallus. Peripheral cells can divide precisely in either anticlinal or periclinal directions to allow radial and circumferential thallus growth (Fig. 2d; Barlow et al. 2002; Brown et al. 1994; Cook 2004; Doty et al. 2014; Marchant and Pickett-Heaps 1973). *Coleochaete soluta* develops in a similar manner, but there are some important distinctions. Peripheral cells are lobed, and discs comprise laterally adherent branched filaments that are contained within a significant layer of mucilage (Fig. 2e; Doty et al. 2014). Conversely, *Coleochaete pulvinata* comprises a 2D branching filamentous thallus, which can differentiate into both prostrate and upright forms, all enclosed within extensive mucilage. Apical growth is exhibited in branches of the upright form, and these are derived from outgrowths of the prostrate form. The Coleochaetophyceae have true multicellular organisation, in which plasmodesmata facilitate intercellular communication. They are also capable of sexual reproduction, in which male (antheridia) and female (oogonia) organs are present although organised differently in discoid (*C. orbicularis*, *C. soluta*) versus cushioned (*C. pulvinate*) forms (Delwiche et al. 2002; Graham et al. 2012; Timme and Delwiche 2010).

The Zygnematophyceae, also known as the ‘conjugating green algae’, represent the most species-rich and morphologically diverse streptophyte lineage. Members of this group lack flagellated cells and instead reproduce by means of conjugation (Wodniok et al. 2011). The Zygnematophyceae are represented by both unicellular and multicellular filamentous forms. Species within the Zygnematophyceae appear to have evolved to lose morphological complexity because they lack both apical growth and plasmodesmata (Fig. 1). The unicellular desmids *Microasterias denticulata* (Holzinger and Lutz-Meindl 2002; Meindl et al. 1994; Neustupa and Stastny 2018) and *Penium margaritaceum* (Domozych 2014; Ochs et al. 2014; Sorensen et al. 2014) have become experimental systems for studies of cell biology and plant cell morphogenesis through ease of their microscopic analysis. *Microasterias denticulata* is highly dissected, bilaterally symmetrical and can reproduce asexually by binary fission (Fig. 2f; Graham et al. 2015). In *P. margaritaceum*, the deposition of new cell wall material at the centre of the cell (‘isthmus’) facilitates bidirectional cell expansion (Fig. 2g). The isthmus also marks the site of cytokinesis, which is facilitated by microtubules (Ochs et al. 2014). The unbranched multicellular and filamentous alga *Spirogyra pratensis* grows by intercalary cell division (Fig. 2h). Filament fragmentation can start growth of new filaments, which can stagnate in freshwater and produce algal blooms very rapidly (Ju et al. 2015; Stancheva et al. 2013).

The Charophyceae comprise large morphologically complex macroalgae, which bear a close resemblance to land plants. The emerging model species *Chara braunii* comprises a central stem, which grows from a terminal apical cell, with branchlets that radiate from axial nodes. Each branchlet within a whorl is derived from a nodal initial and each whorl is separated by an elongated multinucleate internodal cell. Nodal and internodal cells are alternately derived from the apical cell, allowing branchlets to emanate at regular intervals along the stem. Once formed, the resulting branchlets are able to extend (1D growth) and branch (2D growth). *C. braunii* is monoecious and bears both the male (antheridia) and female (oogonia) gametangia on the branchlet nodes (Fig. 2i; Nishiyama et al. 2018).

### The early land pioneers

The earliest pioneers on land were the bryophytes, which include the mosses, liverworts and hornworts. The phylogenetic relationships among the bryophytes and the vascular plants remains a widely debated topic. However, there remains significant support for a monophyletic origin of the bryophytes, and a relationship between the hornworts and a setaphyte clade comprising the mosses and liverworts (de Sousa et al. 2019; Puttick et al. 2018). A unifying feature of the bryophytes is a dominant haploid gametophyte stage of the life cycle, and a diploid sporophyte stage that is both multicellular and nurtured by the gametophyte (Sakakibara et al. 2008).

**Fig. 3:**
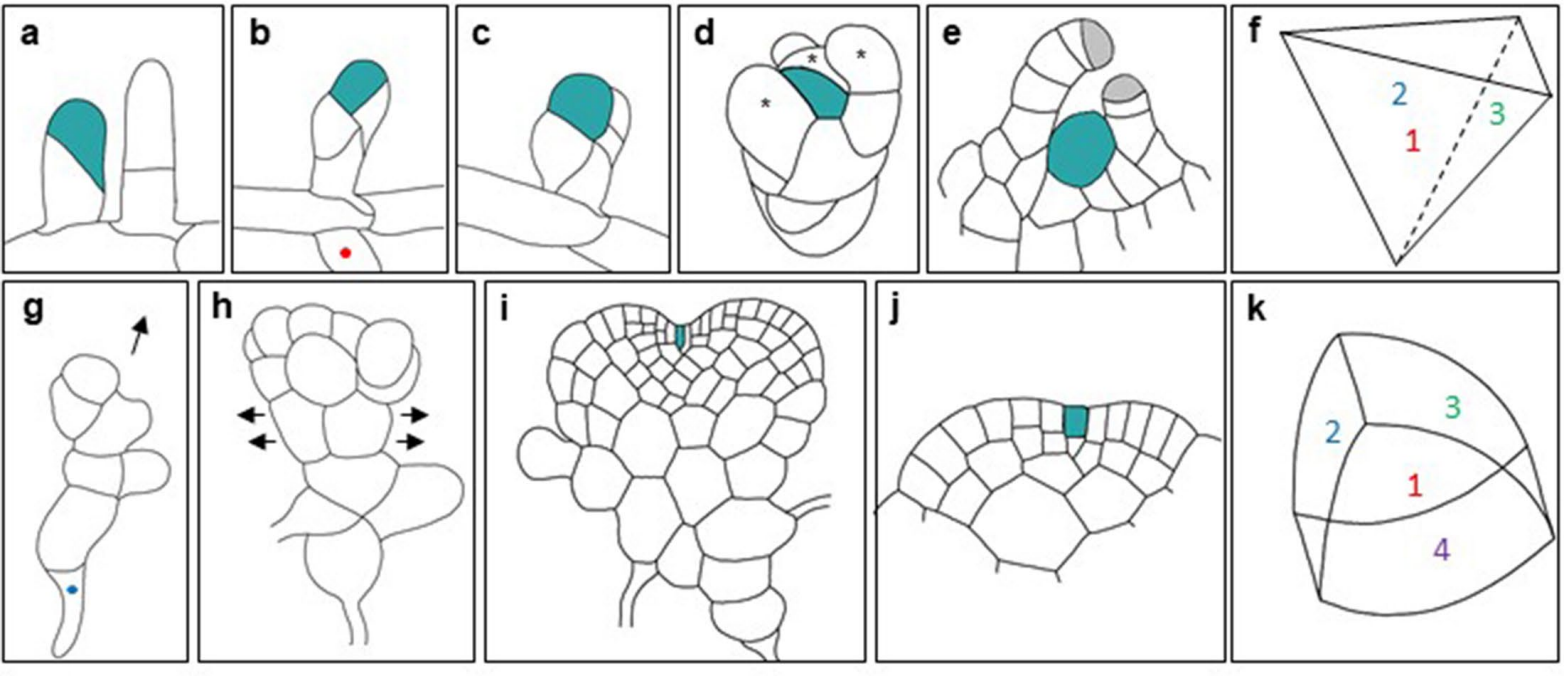
3D growth in land plants. **a**–**f** Schematic diagrams of early gametophore formation in *Physcomitrella patens*, and the establishment of a tetrahedral apical cell with three cutting faces. **a** A gametophore apical cell (left) and a 2D protonemal apical cell (right). Gametophore apical cells swell by probable diffuse growth and then divide obliquely to produce an apical cell (green) and a basal cell. The apical (and basal) cells divide obliquely for a second time (**b**) and an additional two rotating cell divisions take place (**c**) to establish a tetrahedral apical cell with three cutting faces (**d**). Cells derived from the apical cell (denoted by an asterisk) become leaf/phyllid apical cells (*), which form the growing tips (grey) of newly formed phyllids (**e**). **f** Diagrammatic representation of the tetrahedral apical cell with three cutting faces. Images were adapted from Harrison et al. (2009). **g**–**k** Schematic diagrams of early thallus development in *Marchantia polymorpha*, and the establishment of a wedge-shaped apical cell with four cutting faces. **g** A sporeling formed after spore germination under sufficient light conditions. A rhizoid cell (denoted by a blue dot) can be observed. **h** Sporeling cells divide in two planes to produce a branched, spherical thallus. **i** The formation of a lenticular-shaped apical cell (green) allows growth of a unistratose thallus. **j** The lenticular-shaped apical cell becomes incorporated into the apical notch of the growing thallus and transforms into a wedge-shaped apical cell with four cutting faces, allowing cell layers to be added and a multistratose thallus to form. **k** Diagrammatic representation of the wedge-shaped apical cell, which has two lateral faces (1, 2), and one dorsal (3) and one ventral (4) face. Images were adapted from Kny (1890).

The life cycle of the moss *Physcomitrella patens* begins with spore germination and the formation of a chloronemal apical cell, which can divide successively in 1D to enable filament extension. Chloronemal apical cells at growing tips can continue to divide to self-renew or can choose to differentiate into caulonemal apical cells, which can divide to produce filaments of caulonemal cells. Cells that are not immediately adjacent to apical cells can cleave in two planes (2D) to form side branches (a chloronemal apical cell or a caulonemal apical cell) or a gametophore apical cell. Gametophore apical cells divide obliquely twice, and an additional two rotating cell divisions establish a tetrahedral apical cell with three cutting faces, thus establishing three-dimensional (3D) growth (Fig. 3a–e). Self-renewal and differentiation processes produce a leafy gametophore composed of many phyllids spirally arranged around a stem. Successive anticlinal and periclinal divisions distal to growing tips allow phyllids to assume their final shape. Mature gametophores bear the reproductive organs antheridia and archegonia (Harrison et al. 2009).

The filamentous phase of the liverwort *Marchantia polymorpha* is more transient than that of *P. patens*. In sufficient light conditions, spore germination gives rise to sporelings that grow in an isotropic manner before establishing an apical cell. However, in low red-light conditions, sporelings can divide unidirectionally (1D) to form a polarised ‘protonema-like’ structure (Ishizaki et al. 2016; Nishihama et al. 2015). Cells of the sporelings then divide in a second plane to produce a branched, spherical thallus. A lenticular-shaped apical cell with two cutting faces is established, which subsequently divides to produce a thallus comprising a single cell layer (Shimamura 2016). The apical cell becomes incorporated into an apical notch and ultimately adopts a distinctive ‘wedge-shaped’ morphology with the capacity to divide in four planes (3D growth) and add cell layers to build a multistratose thallus that can anchor to substrata (Fig. 3f–i; Crandall-Stotler 1981; O’Hanlon 1926). *Marchantia polymorpha* can reproduce asexually by fragmentation, by producing gemmae in gemma cups (‘splash cups’), or sexually by producing antheridia and archegonia on free-living dioecious plants (Miller 1964; Miller and Colaiace 1969).

## The brown algae

**Fig. 4:**
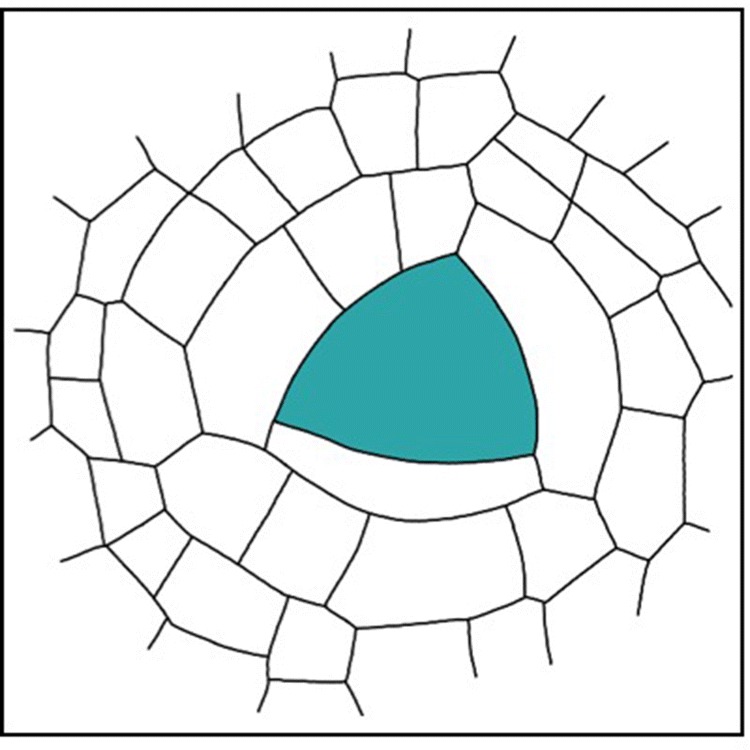
3D growth in brown algae. Schematic diagram showing the apical cell (green) of the brown alga *Turbinaria turbinata*, which has three cutting faces. Image adapted from Blomquist (1945).

The brown algae are a group of multicellular marine algae that have evolved independently of land plants for more than one billion years. Yet brown algae and land plants share traits that are remarkable similar. These include apical growth, the formation of plasmodesmata for intercellular communication and the development of a multicellular sporophyte generation (Arun et al. 2013, 2019; Terauchi et al. 2015). Most notably, families within the Fucales exhibit 3D growth and produce apical cells with three cutting faces (Fig. 4). The Sargassum group has been rather comprehensively studied because members of this group can arrange organs radially around a central axis (Blomquist 1945; Fritsch 1945). In *Sargassum muticum* (and other species such as *S. vulgare*), an apical cell, which is contained within an apical pit filled with mucilage, divides from all three faces to produce promeristematic cells. These repeatedly divide to give rise to cells that make up the meristoderm, cortex and medulla layers of the alga body (Kaur 1999; Linardić and Braybrook 2017; Peaucelle and Couder 2016). *Sargassum muticum* comprises a root-like holdfast, which gives rise to the central axis (stipe). The stipe has a meristematic apex that produces hierarchically organised primary branches, each able to produce radially organised leaf-like blades, air bladders and reproductive receptacles from its own meristematic apex (Linardić and Braybrook 2017; Peaucelle and Couder 2016).

## Summary

There are important morphological distinctions that distinguish the Charophytes from land plants. Most notably, Charophyte body plans are derived from growth in 1D or 2D and comprise filaments, mats and branches. However, land plants acquired apical cells that could cleave in three or more planes, enabling them to develop the morphological toolkit required to survive out of the water. 3D growth is thus an invariable and pivotal feature of all land plants. Intriguingly, 3D apical growth has also evolved within some species of brown algae, which have been evolving independently of the Streptophytes for over a billion years.
